# MYC-regulated pseudogene HMGA1P6 promotes ovarian cancer malignancy via augmenting the oncogenic HMGA1/2

**DOI:** 10.1038/s41419-020-2356-9

**Published:** 2020-03-03

**Authors:** Xiaoxue Tian, Jianping Song, Xiyu Zhang, Mingyao Yan, Shourong Wang, Yuqiong Wang, Limei Xu, Ling Zhao, Jian-jun Wei, Changshun Shao, Beihua Kong, Zhaojian Liu

**Affiliations:** 1 Department of Obstetrics and Gynecology, Qilu Hospital, Shandong University, 107 Wenhua Xi Road, 250012 Jinan, Shandong Province China; 2 Key Laboratory of Experimental Teratology, Ministry of Education, Department of Cell Biology, Shandong University School of Medicine, 44 Wenhua Xi Road, 250012 Jinan, Shandong Province China; 3 Department of Molecular Medicine and Genetics, Shandong University School of Medicine, 250012 Jinan, Shandong China; 4 Department of Obstetrics & Gynecology, Peking University First Hospital, 100034 Beijing, China; 5 Department of Pathology, Northwestern University School of Medicine, Chicago, IL USA; 6 Institutes for Translational Medicine, State Key Laboratory of Radiation Medicine and Protection, Soochow University, 215123 Suzhou, Jiangsu Province China

**Keywords:** Gynaecological cancer, Translational research

## Abstract

Pseudogenes have long been considered as nonfunctional genomic sequences. Recent studies have shown that they can potentially regulate the expression of protein-coding genes and are dysregulated in diseases including cancer. However, the potential roles of pseudogenes in ovarian cancer have not been well studied. Here we characterized the pseudogene expression profile in HGSOC (high-grade serous ovarian carcinoma) by microarray. We identified 577 dysregulated pseudogenes and most of them were up-regulated (538 of 577). HMGA1P6 (High mobility group AT-hook 1 pseudogene 6) was one of the overexpressed pseudogenes and its expression was inversely correlated with patient survival. Mechanistically, HMGA1P6 promoted ovarian cancer cell malignancy by acting as a ceRNA (competitive endogenous RNA) that led to enhanced HMGA1 and HMGA2 expression. Importantly, HMGA1P6 was transcriptionally activated by oncogene MYC in ovarian cancer. Our findings reveal that MYC may contribute to oncogenesis through transcriptional regulation of pseudogene HMGA1P6 in ovarian cancer.

## Introduction

Ovarian cancer is the most lethal gynecologic cancer. Globally, there are 239,000 new cases (3.6% of all cancer cases) and 152,000 deaths reported annually^1^. More than 75% of affected women are diagnosed at an advanced stage and there’s no effective screening strategy for early diagnosis^2^. The 10-year survival is <30% and has not been improved significantly over the last 30 years^3^. HGSOC is the most common histologic subtype, accounting for three quarters of ovarian cancer^4^. Standard treatments for newly diagnosed cancer consist of cytoreductive surgery and platinum-based chemotherapy. PARP (Poly ADP-ribose polymerase) inhibitors have recently been demonstrated a significant therapeutic effect in recurrent HGSOC with HRD (homologous recombination defects). Unfortunately, like many targeted therapies, the response of most patients to PARP inhibitors is transient. The causes of PARP resistance in patients remain to be elucidated^5^. There are currently no approved immune therapies for ovarian cancer^6^. The mechanisms in controlling ovarian cancer progression and recurrence are probably complex and remain to be fully characterized.

Pseudogenes have long been labeled as ‘junk DNA’ which are faulty copies of genes that arise during the evolution of genome^7^. The human genome is estimated to contain more than 18,000 pseudogenes, two thirds of which are transcribed^8^. Accumulating evidence showed that pseudogenes exhibit functional roles. Pseudogenes can regulate gene expression at transcriptional and post-transcriptional level. Pseudogene PTENP1 is biologically active as it can regulate PTEN by acting as a decoy for PTEN-related miRNAs^9^. A large number of pseudogenes are dysregulated in various types of cancer including ovarian cancer, suggesting their potential role in cancer^10^. The BRAF pseudogene acts as a ceRNA and induces lymphoma in vivo^11^. PTTG3P promotes cell growth and metastasis via up-regulating PTTG1 and activating PI3K/AKT signaling in hepatocellular carcinoma (HCC)^12^. PDIA3P1 is upregulated and acts as an oncogene in HCC^13^. Pseudogene DUXAP8 promotes non-small-cell lung cancer cell proliferation and invasion by epigenetically silencing EGR1 and RHOB^14^. Overexpression of HMGA1P6 could contribute to increase HMGA1 levels in human pituitary tumors^15^. The expression patterns and potential roles of pseudogenes in ovarian cancer remain unclear.

In this study, we identified the differentially expressed pseudogenes through transcriptomic data analysis in HGSOC compared to normal tissues. We further demonstrated that HMGA1P6 was one of highly expressed pseudogenes in HGSOC which promoted ovarian cancer aggressiveness through modulating HMGA1/2. Importantly, HMGA1P6 was a direct transcriptional target of MYC, which is the most frequently amplified gene in HGSOC.

## Materials and methods

### Patients and tissue samples

Ovarian cancer and fallopian tube tissues were collected from Department of Obstetrics and Gynecology, Qilu Hospital, Shandong University from April 2009 to July 2015. The HGSOC specimens were from primary ovarian cancer patients with no surgery or chemotherapy previously. Fallopian tube tissues were from patients who received a total hysterectomy and bilateral salpingo-oophorectomy for uterine diseases or benign neoplastic adnexal pathologic changes. Ethics Committee of Shandong University approved the study and all participants gave written informed consent.

### Tissue preparation and RNA extraction

Fresh tissue samples were collected within 2 h of surgery. All fresh tissues were sliced to 5 mm^3^ in size and immersed in 10 vol of RNALater (Ambion, Austin, TX). The tissue samples were then stored in a −80 °C laboratory freezer. RNA was isolated using TRIzol reagent (Invitrogen) according to the manufacturer’s protocol.

### Microarray analysis

Six samples of each FT, LGSOC and HGSOC tissues microarray analysis were conducted (Human lncRNA Array v2.0 8 × 60K). The arrays were scanned by the Agilent Scanner G2505B. Agilent Feature Extraction software (version 11.0.1.1) was used to analyze acquired array images. Quantile normalization and subsequent data processing were performed using the GeneSpring GX v12.1 software package (Agilent Technologies). The microarray data generated in this study have been deposited in NCBI GEO database under the accession number GSE135886.

### Bioinformatics analysis

Pseudogenes of microarray data were annotated by GENCODE v.18. TCGA and GTEx normal RNA-Seq data were downloaded from UCSC Xena (https://xenabrowser.net/). R-3.0.1 (http://www.r-project.org/) was used for dysregulated pseudogene hierachical cluster, heatmap visualization and Pearson correlations analysis. GraphPad Prism was used for survival analysis. Kaplan–Meier plots were tested by Log-rank tests.

### Cell culture and treatment

HO8910 and HEK293T cell lines were purchased from China Type Culture Collection (Shanghai, China). A2780, HEY, SKOV3 cell lines were from Jian-Jun Wei’s lab. HEY, HO8910, and A2780 cells were cultured in DMEM medium (Gibco, Invitrogen). SKOV3 cells were cultured in McCoy’s 5A medium. All media contained 10% FBS (Gibco, Grand Island, NY, USA). Doxycycline Hydrochloride (Sangon Biotech, Shanghai, China) was added to cell culture medium at the final concentration of 100 ng/ml for 48–72 h when induced expression of tet-on shRNA vector is needed. All cells were cultured at 37 °C with 5% CO_2_ in a humidified incubator.

### RNA isolation and qPCR

Total RNA was extracted from cells or fresh tissues with Cell Total RNA Isolation Kit (Foregene, Chengdu, China) following manufacturer’s instructions. cDNA was synthesized by using PrimeScript RT reagent Kit (Takara, Japan). qPCR was performed using SYBR Green mix and detected by the Bio-Rad CFX96. Primers information used in this study were listed in Supplementary Table 1.

### Transient transfection

Plasmids, siRNAs, miRNA mimics and inhibitors were transiently transfected into cells using jetPRIME transfection reagent (PolyPlus, Illkirch, France) following manufacturer’s instructions. RNA and proteins were extracted after 48 or 72 h transfection. Sequences of siRNAs, miRNA mimics and inhibitors were showed in Supplementary Table 2.

### Lentiviral vector construction, viral production and infection

HMGA1P6 transcript sequence was cloned into pcDNA3.1 and pGIPZ vectors. Tet-on-sh-HMGA1P6 shRNA vector (pZIP-TRE3G-ZsGreen-Puro) was constructed by Vigene Biosciences (Jinan, Shandong, China) based on si-HMGA1P6 sequence. Those lentiviral vectors, along with pMD2.G and psPAX2, were co-transfected into HEK293T cells for lentivirus production. Cells were infected with lentivirus for 24–48 h and selected for 2 weeks in medium containing 2 μg/ml puromycin (Merck Millipore, Billerica, MA, USA) to acquire stable expression cells.

### Western blotting

The samples were lysed on ice in RIPA buffer (Beyotime Institute of Biotechnology, China) and protein concentration was determined using the BCA assay kit (Beyotime Institute of Biotechnology, China). Protein samples were separated by SDS–PAGE and electro-transferred onto PVDF membrane. The membrane was blocked in 5% skimmed milk and incubated overnight with primary antibodies at 4 °C. Proteins of interest were detected with appropriate HRP-labeled secondary antibodies and developed using ECL system (GE Healthcare, UK). All antibody information was listed in Supplementary Table 3.

### EdU cell proliferation assay

Cells were seeded on glass coverslips in 24-well plates at densities of 4–10 × 10^4^ cells per well. EdU assay was performed using EdU Kit (RiboBio, Guangzhou, China) following manufacturer’s instruction. Briefly, cells was incubated in cell culture medium containing EdU for 0.5–1 h. Then the cells were fixed and then stained by Apollo fluorescent dye and Hoechst33342. Then the glass coverslips were fixed on glass slide and captured under a fluorescent microscopy.

### MTT assay

Cells were seeded in 96-well plates in triplicate at densities of 0.5–1 × 10^3^ cells per well. Cell proliferation was monitored every day for next 5 days. After incubation at designated times, 10 μl MTT reagent (5 mg/ml) was added to each well and continued to incubate for 4 h at 37 °C. Then supernatant was discarded and 100 μl of DMSO was added to each well. The absorbance at 562 nm was measured using a microplate reader (Bio-Rad, Hercules, CA, USA). All experiments were repeated at least three times.

### Clonogenic assay

The cells were seeded in a six-well plate (500–2000 cells per well) and cultured for 1–2 weeks. The colonies were fixed with methanol, and stained with 0.1% crystal violet, and the number of clonies containing more than 50 cells was counted. The data presented are the mean ± S.E. and represents three independent experiments.

### Immunohistochemistry

Formalin-fixed and paraffin-embedded tissues were sectioned at 4 μm. Tissue slides were deparaffinized in xylene and rehydrated in a graded series of ethanol. Antigen retrieval was performed in EDTA by microwave heating. Nonspecific antigens were blocked with 1.5% normal goat serum. Primary antibody for HMGA1 and Ki-67 (1:100 dilution) was incubated on the slides overnight at 4 °C. Then the slides were incubated with secondary antibody and sections were then stained with diaminobenzidine. IHC slides were reviewed by two pathologists in a blinded manner.

### 3D cell culture

Spheroid formation of ovarian cancer cells was established in 24-well VitroGel 3D-RGD (The Well Bioscience, North Brunswick, USA) culture plates according to the manufacturer’s protocol. Cell culture medium was changed every other day. The spheroids were harvested after 10 days culture and spheroids were imaged under a light microscope.

### Matrigel invasion assay

Matrigel invasion assay was performed in 24-well matrigel coated chambers with 8 μm pores (BD falcon, Bedford, MA, USA). The matrigel was 1:10–1:15 diluted with no FBS DMEM. 200 µl cell suspension containing 10–15 × 10^4^ cells were plated into the upper chambers in medium containing no FBS, and lower chambers were filled with 700 μl culture media containing 30% FBS as a chemo-attractant. The chambers were incubated at 37 °C for 4–48 h depending on different cell lines. Successfully invaded cells were fixed with methanol and stained with 0.1% crystal violet for 15 min, and counted under a light microscope.

### Quantitation of cell migration by High-content Image analysis

Cells were seeded in 96-cell plate (5000 cells per well) and cultured for 24 h. Then cells were imaged with an Operetta high-content imaging system (PerkinElmer) at ×10 magnification. DPC (Digital Phase Contrast) algorithm was used to take images for 7 h at the time interval of 10 min. Then Harmony software were used to analyze the displacement property of cell migration.

### Luciferase assay

For 3′UTR sensor luciferase assay, HMGA1P6 transcript and HMGA1/2 3′UTR were cloned into pmirGLO vector (Promega). Mutant constructs were generated by overlap extension PCR. Vectors and microRNAs were co-transfected into HEK293T cells and luciferase activity was measured 48 h after transfection using Dual-Glo Luciferase Assay System (Promega). For promoter reporter assay, HMGA1P6 promoter region was cloned into pGL4.26 plasmids. Then HMGA1P6 promoter report vector was co-transfected with si-MYC or penter-MYC plasmids into HEK293T cells and luciferase activity was measured as described.

### RIP assay

RIP (RNA Immunoprecipitation) assay was performed using EZ-Magna RIP RNA-Binding Protein Immunoprecipitation Kit (Merck KGaA, Darmstadt, Germany) following manufacturer’s instructions. Briefly, cells were lysed with RIP lysis buffer and incubated with magnetic beads coated with anti-Ago2 antibody (Merck) and IgG (Millipore) was used as a negative control. After that, qRT-PCR was performed to detect the enrichment of HMGA1P6 and miRNAs.

### RNA pull-down assay

Biotin‐labeled HMGA1P6 transcripts was obtained using in vitro transcription with T7 RNA polymerase from the TranscriptAid T7 High Yield Transcription Kit (Thermo Fisher Scientific). RNA pull-down was performed using the Magnetic RNA-Protein Pull-Down kit (Thermo Fisher Scientific, USA) according to manufacturer’s protocol. The protein bands interest on the gel were lysed and identified by mass spectrometry (MS) and confirmed by western blotting.

### ChIP assay

ChIP (chromatin immunoprecipitation) assay was conducted using EZ-Magna ChIP A/G Chromatin Immunoprecipitation Kit (Merck KGaA, Darmstadt, Germany). Cells were cross-linked using 37% formaldehyde. Then the cells were lysed in lysis buffer and DNA was sheared to ~200–500 bp fragments by sonication. The cell lysis was incubated with anti-MYC antibody and magnetic beads overnight at 4 °C with rotation. DNA was purified and analyzed by qRT-PCR.

### Nude mice xenografts

Female BALB/c-nude mice (6–8-week old, 5 mice for each group) were randomly divided into two groups and were injected subcutaneously with ovarian cancer cells with HMGA1P6 overexpression or knockdown compared to control group as previously described^16^. The Shandong University Animal Ethics Research Board approved all animal procedures.

### Statistics analysis

Student’s *t*-test and one-way ANOVA analysis were used to determine significance. Chi-square test was used to analyze the differences of clinical characteristics. Results represent the mean ± S.D. of three independent experiments. Statistically significance was considered as: *p < 0.05, **p < 0.01.

## Result

### Transcriptome analysis of aberrantly expressed pseudogenes in HGSOCs

**Fig. 1:**
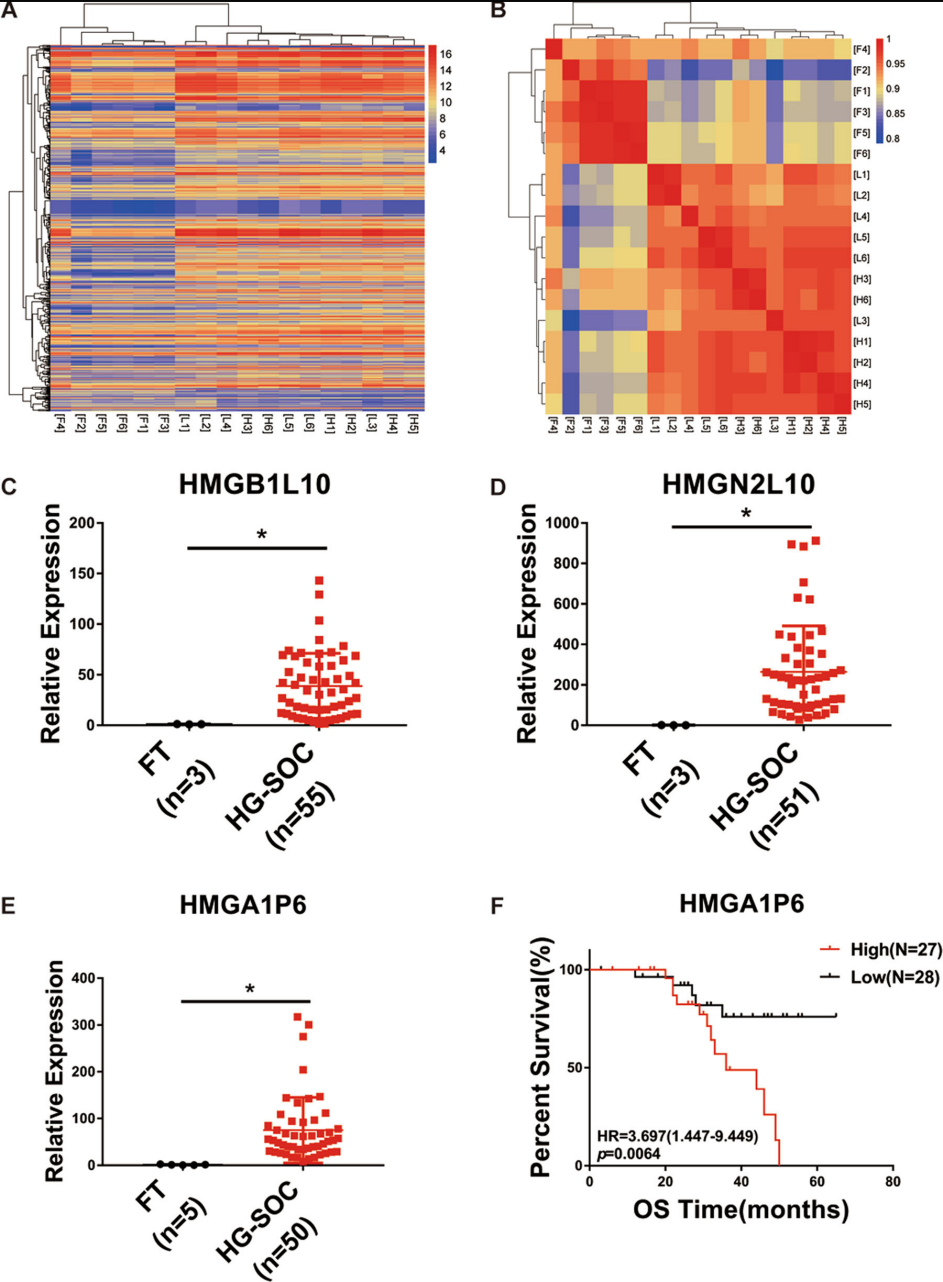
Transcriptome analysis and validation of dysregulated pseudogenes in HGSOCs. **a** Hierarchically clustered heatmaps of 577 dysregulated pseudogenes in HGSOCs (log2 fold change >1 or <−1). **b** Correlation matrix of all expressed pseudogenes (*n* = 1296) in HGSOCs (*n* = 6) and LGSOCs (*n* = 6) comparing with FTs (*n* = 6). **c**–**e** Validation of selected dysregulated pseudogenes (HMGN2L10, HMGB1L10, and HMGA1P6) in HGSOCs detected by qPCR. **f** Overall survival (OS) of HMGA1P6 in high-expression group (*n* = 27) vs. low-expression (*n* = 28) group. *P* value was obtained by Log-rank test. Data are presented as means ± S.D. *p < 0.05.

To identify pseudogenes specifically associated with HGSOC, we performed transcriptome analysis on HGSOC compared with LGSOC (low-grade serous ovarian carcinoma) and normal FT (fallopian tube) using Human lncRNA array V2.0 (8 × 60 K, Agilent). We identified 577 dysregulated pseudogenes in HGSOCs relative to FTs (Fig. 1a and Supplementary Table 4). Interestingly, the majority of dysregulated pseudogenes were up-regulated (538 of 577). Hierarchical cluster and correlation matrix heat map analysis showed that HGSOC exhibited specific pseudogene expression pattern compared to normal tissues (Fig. 1a, b). We next validated three pseudogenes that were upregulated in HGSOCs. As shown in Fig. 1c–e, HMGB1L10, HMGN2L10, and HMGA1P6 were upregulated in HGSOCs compared to FTs. Among these three pseudogenes, HMGA1P6 has been reported to be overexpressed in human anaplastic thyroid and pituitary tumors^15,17^. TCGA data analysis showed that HMGA1P6 was overexpressed in stomach adenocarcinoma (Supplementary Fig. 1a). Therefore, HMGA1P6 was selected for further investigation. We then evaluated the correlation between HMGA1P6 expression and the clinical outcome in HGSOCs by Kaplan–Meier method. Notably, patients with high expression level of HMGA1P6 had a shorter overall survival time than those with low expression did (Fig. 1f). Association analysis between HMGA1P6 expression level and other clinicopathological factors were showed in Supplementary Table 5. These data indicate that pseudogene expression pattern might be a signature of HGSOC and pseudogenes such as HMGA1P6 play a role in pathogenesis of HGSOC.

### HMGA1P6 promotes ovarian cancer cell proliferation and xenograft tumor growth

**Fig. 2:**
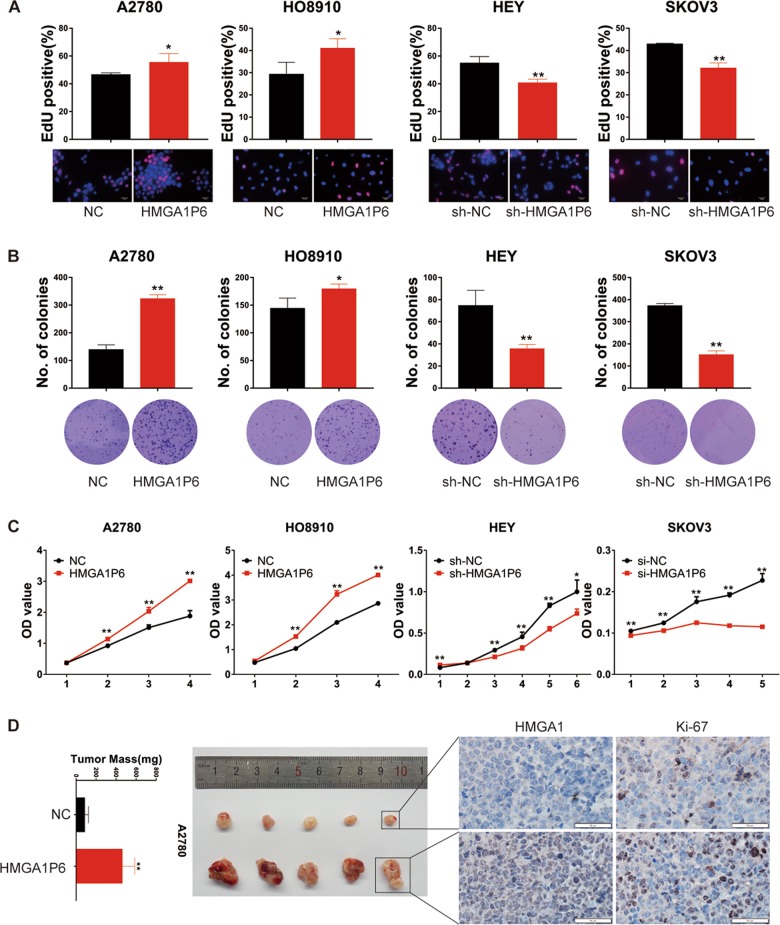
HMGA1P6 promotes ovarian cancer cell proliferation and tumor xenograft growth. **a**–**c** EdU, clonogenic and MTT assays were performed to measure the effect of HMGA1P6 on ovarian cancer cell proliferation. **d** Representative tumors in xenografts of A2780 cells with HMGA1P6 overexpression compared to control cells (left panel), immunohistochemical staining of Ki-67 and HMGA1 were performed in tumor tissues (right panel). Data are presented as means ± S.D. *p < 0.05, **p < 0.01.

To explore the biological function of HMGA1P6 in ovarian cancer pathogenesis, we established stable cell lines with HMGA1P6 overexpression or knockdown. The overexpression and knockdown efficiency were detected by qPCR as illustrated in Supplementary Fig. 1b. We then conducted EdU (5-Ethynyl-2′-deoxyuridine) assay and found HMGA1P6 overexpression significantly increased the number of EdU-positive cells, while knockdown of HMGA1P6 decreased the ratio of EdU-positive cells compared with control group (Fig. 2a). In accordance with EdU data, clonogenic assay showed that ectopic expression of HMGA1P6 enhanced clonogenic capacity in A2780 and HO8910 cells whereas knockdown of HMGA1P6 significantly reduced the colony-forming efficiency in HEY and SKOV3 cells (Fig. 2b). Moreover, growth curve analysis showed that HMGA1P6 overexpression dramatically enhanced the proliferation of ovarian cancer cells, while silencing HMGA1P6 induced an opposite effect (Fig. 2c). To further investigate the tumorigenic effects of HMGA1P6 on ovarian cancer cells in vivo, a subcutaneous xenograft model was used and both the volumes and weights of the tumors in HMGA1P6 overexpression group were remarkably larger than control group while HMGA1P6 knockdown significantly decreased tumor volume and weight (Fig. 2d, Supplementary Fig. 1c, d). Additionally, the number of Ki-67 positive cells was significantly increased in tumors with HMGA1P6 overexpression (Fig. 2d). These findings demonstrate that HMGA1P6 facilitated ovarian cancer cell proliferation in vitro and promoted tumor growth in vivo.

### HMGA1P6 enhances sphere formation efficiency and invasiveness of ovarian cancer cells

**Fig. 3:**
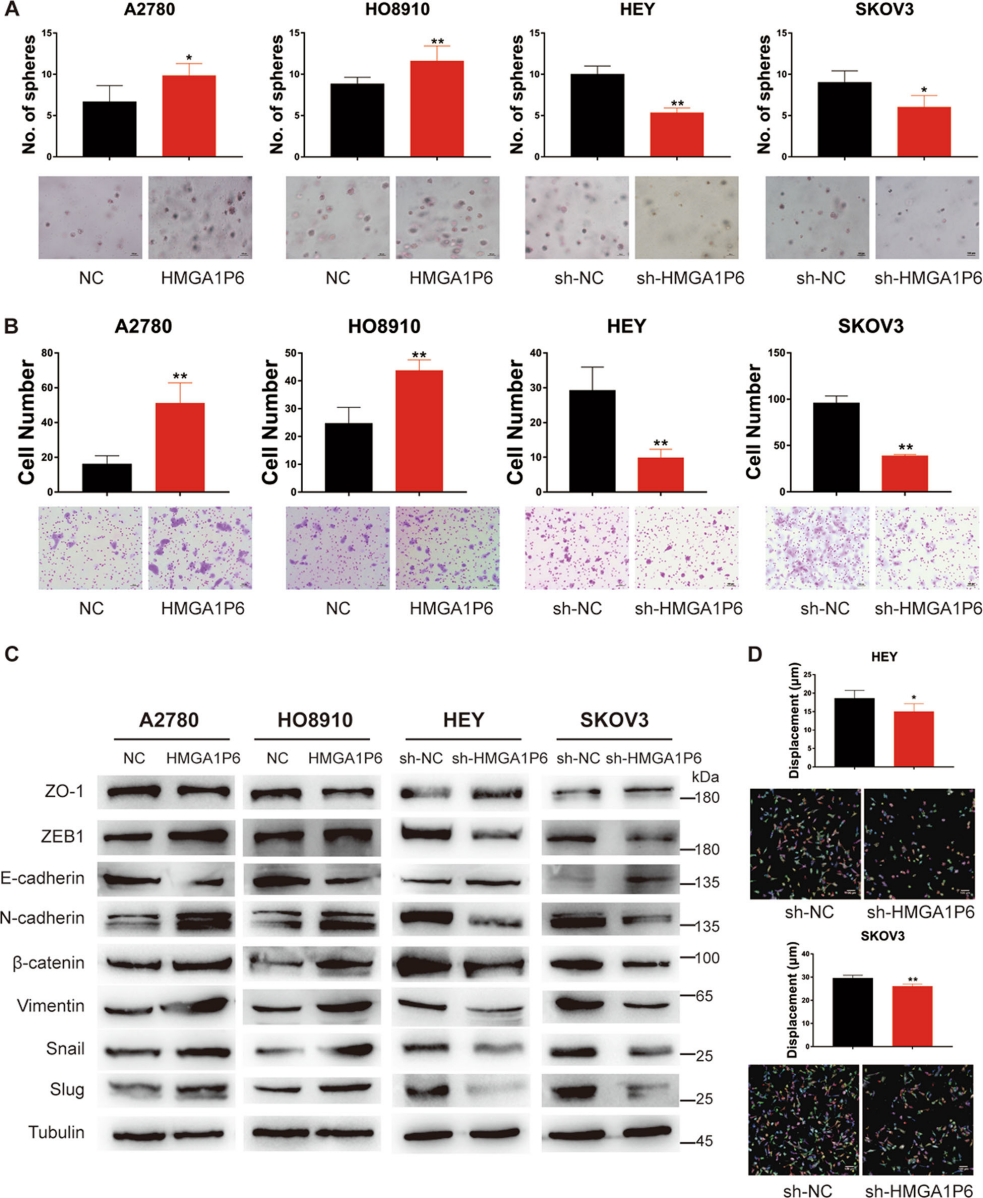
HMGA1P6 enhances sphere formation efficiency and invasiveness of ovarian cancer cells. **a**, **b** 3D culture and matrigel invasion assay were conducted in ovarian cancer cell lines with HMGA1P6 overexpression or knockdown. **c** Western blot analysis of EMT related markers in ovarian cancer cells with HMGA1P6 overexpression or knockdown. **d** High-content Digital imaging assay was carried out to analyze cell migration of HEY and SKOV3 cells with HMGA1P6 knockdown. Migration distance was tracked and recorded. Data are presented as means ± S.D. *p < 0.05, **p < 0.01.

To further explore the oncogenic potential of HMGA1P6 in ovarian cancer, 3D (three-dimension) cell culture were conducted to evaluate the effect of HMGA1P6 on sphere formation efficiency. As demonstrated in Fig. 3a, ectopic expression of HMGA1P6 significantly enhanced the sphere formation efficiency in A2780 and HO8910 cells compared to control cells while HMGA1P6 knockdown markedly suppressed sphere formation. Western blot analysis showed that OCT4, KLF4, SOX2, and NANOG were downregulated in HEY and SKOV3 cells with HMGA1P6 knockdown (Supplementary Fig. 1e). In addition, ectopic expression of HMGA1P6 enhanced the invasive capacity whereas knockdown of HMGA1P6 decreased invasion potential in ovarian cancer cells (Fig. 3b). Not surprisingly, forced expression of HMGA1P6 upregulated the levels of mesenchymal markers and reduced the levels of epithelial markers. On the contrary, knockdown of HMGA1P6 reversed epithelial–mesenchymal transition (Fig. 3c). Moreover, we measured the effect of HMGA1P6 on ovarian cancer cell migration by a high-content imager (Perkin Elmer) and analyzed by harmony software. As expected, HMGA1P6 knockdown dramatically reduced migration distance of HEY and SKOV3 cells compared to control cells (Fig. 3d). The Warburg effect plays a vital role in promoting tumor initiation and progression^18^. We next tested the effect of HMGA1P6 on aerobic glycolysis in ovarian cancer cells. As a result, ectopic expression of HMGA1P6 increased glycolytic activity by the measurement of the ECAR (extracellular acidification rate). Conversely, HMGA1P6 knockdown led to decreased glycolytic activity and ATP production (Supplementary Fig. 2a, b). Taken together, these data suggest that HMGA1P6 exhibit oncogenic potential in ovarian cancer.

### HMGA1P6 modulates HMGA1 and HMGA2 expression in ovarian cancer

**Fig. 4:**
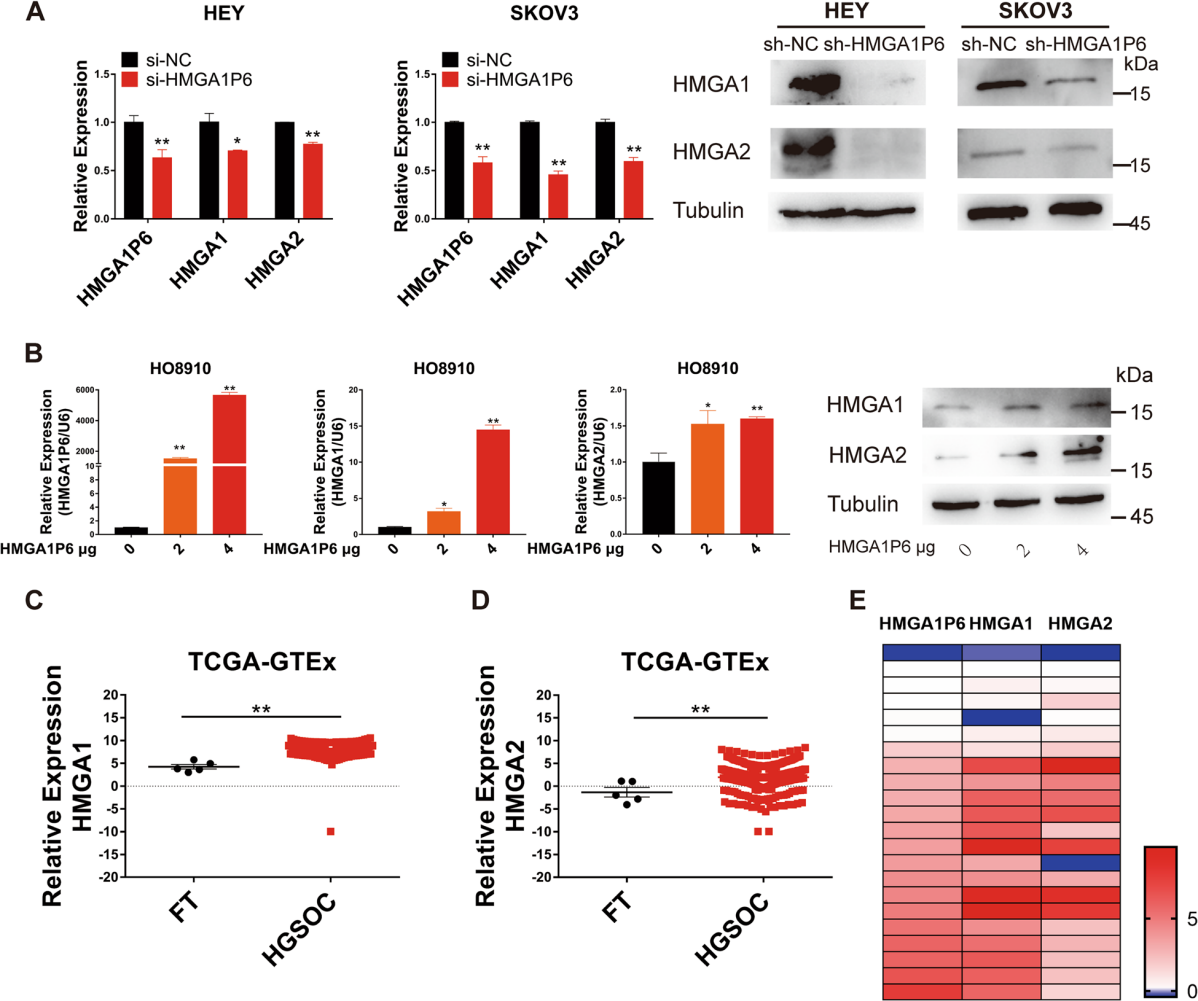
HMGA1P6 positively regulates HMGA1/2 expression in ovarian cancer. **a** Expression of HMGA1P6, HMGA1/2 mRNA (left two panels) and protein (right two panels) level was measured in HEY and SKOV3 cells with HMGA1P6 knockdown. **b** Expression of HMGA1P6, HMGA1/2 mRNA (left panel) and HMGA1/2 protein (right panel) level was measured in HO8910 cells transfected with HMGA1P6 in gradient. **c**, **d** Relative expression of HMGA1 and HMGA2 was analyzed in ovarian cancer TCGA-GTEx data. **e** Association analysis of HMGA1P6, HMGA1 and HMGA2 expression in HGSOC tissues (*n* = 24). Data are presented as means ± S.D. *p < 0.05, **p < 0.01.

It has been reported that both HMGA1 and HMGA2 are high expressed in HGSOC and correlated with tumor progression and poor prognosis^19–22^. We postulated that pseudogene HMGA1P6 could modulate the expression of HMGA1 and HMGA2. We first measured the expression level of HMGA1 and HMGA2 with HMGA1P6 knockdown. As expected, both mRNA and protein level of HMGA1 and HMGA2 were significantly reduced in HEY and SKOV3 cells with HMGA1P6 knockdown compared to control cells (Fig. 4a). We next transfected gradient concentration of HMGA1P6 plasmid into ovarian cancer cells and then evaluated HMGA1 and HMGA2 expression. As shown in Fig. 4b, both mRNA and protein level of HMGA1 and HMGA2 were upregulated in HO8910 cells with HMGA1P6 overexpression. Additionally, TCGA and GTEx data analysis showed that both HMGA1 and HMGA2 were commonly overexpressed in HGSOCs compared to FTs (Fig. 4c, d). Heatmap of qPCR data showed that HMGA1P6 was correlated with HMGA1/2 in HGSOCs (Fig. 4e). These data support that HMGA1P6 modulates HMGA1 and HMGA2 expression in ovarian cancer.

### HMGA1P6 regulates HMGA1/2 as a competing endogenous RNA

**Fig. 5:**
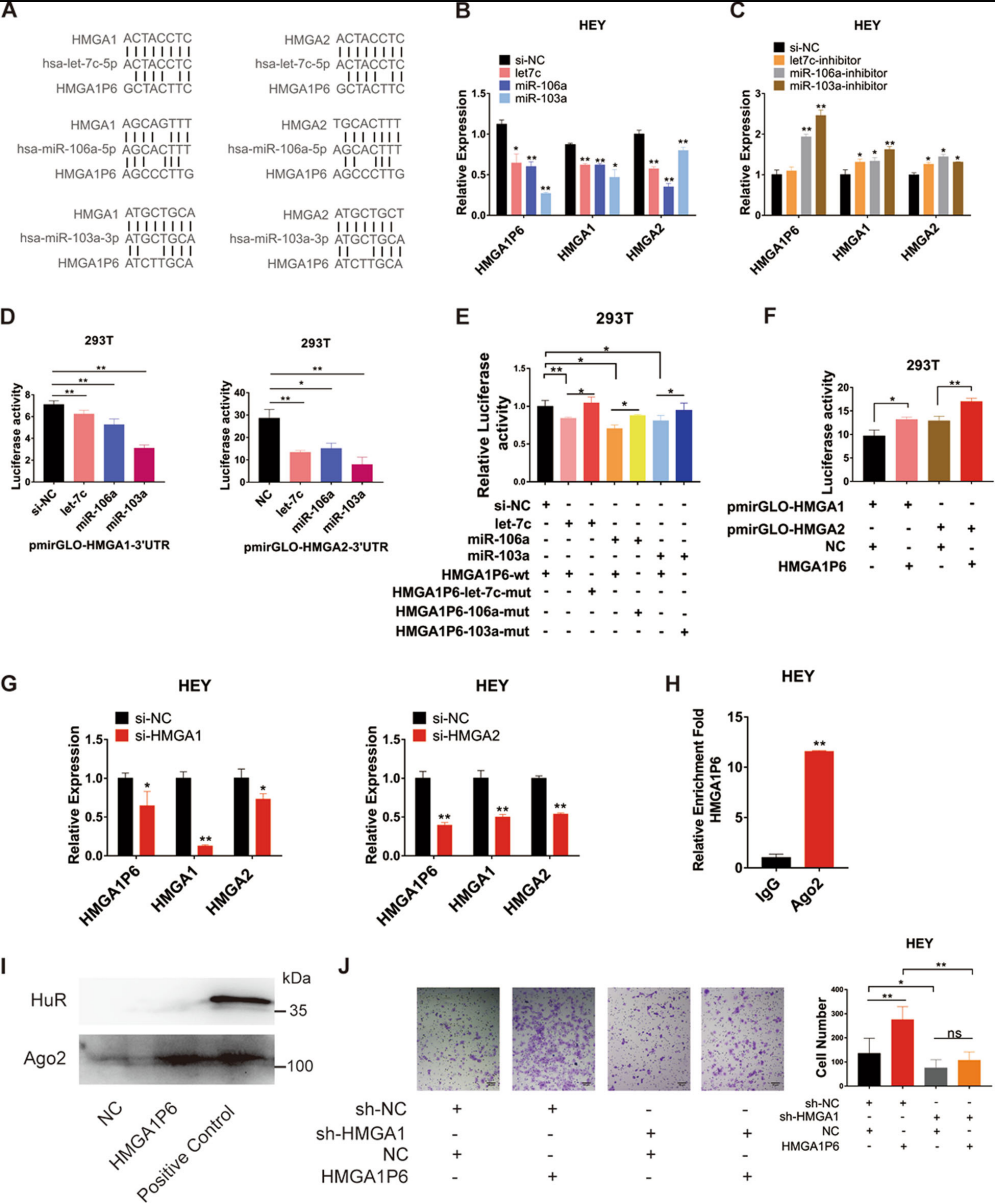
HMGA1P6 regulates HMGA1/2 as a competing endogenous RNA. **a** Bioinformatics analysis of potential miRNAs that target HMGA1P6, HMGA1 and HMGA2 3′UTR was conducted. **b**, **c** HMGA1P6, HMGA1 and HMGA2 expression were measured by qPCR after transfection of miRNA mimics or inhibitors. **d**–**f** Luciferase activity was tested after co-transfection of reporter plasmid containing HMGA1/2 3′UTR, HMGA1P6 wild type or mutant transcript and miRNA mimics. **g** Relative expression of HMGA1P6 and HMGA1/2 was measured in HEY cells after transfection of HMGA1/2 siRNAs. **h** Relative enrichment fold change of HMGA1P6 was analyzed in Ago2-RIPs compared to control IgG-RIPs. **i** Ago2 in biotinylated HMGA1P6 pulldown lysates in HEY cells was tested by western blot. **j** Matrigel invasion assay was performed in HEY cells transfected with different vectors for rescue experiment. Data are presented as means ± S.D. *p < 0.05, **p < 0.01.

Pseudogene RNA can compete with the parental gene RNA for miRNAs and thereby influence gene expression^23^. Pseudogene PTENP1 is reported to upregulated expression level of PTEN by competing for binding to miRNAs shared with PTEN^9^. We hypothesized that HMGA1P6 may regulates HMGA1 and HMGA2 through ceRNA mechanism since they have highly sequence similarity. We first analyzed the subcellular localization of HMGA1P6 with PCR after cellular fractionation and HMGA1P6 was shown to distribute in both nucleus and cytoplasm (Supplementary Fig. 2c). Bioinformatics analysis by targetscan and miRDB revealed that three miRNAs (hsa-let-7c-5p, hsa-miR-106a-5p and hsa-miR-103a-3p) had complementary sequence pairing with HMGA1P6, HMGA1, and HMGA2 (Fig. 5a). Transfection mimics of these three miRNAs significantly inhibited the expression level of HMGA1P6, HMGA1 and HMGA2 (Fig. 5b). Conversely, miRNA inhibitors enhanced HMGA1P6, HMGA1, and HMGA2 expression (Fig. 5c). Next step, we conducted luciferase assay and found that transfection mimics of these three miRNAs obviously reduced the luciferase activity of pmirGLO-HMGA1/2–3′UTR (Fig. 5d). Consistently, miRNA mimics significantly inhibited luciferase activity of wild type but not mutant pmirGLO-HMGA1P6 (Fig. 5e). Overexpression of HMGA1P6 could enhanced luciferase activity of pmirGLO-HMGA1/2 3′UTR (Fig. 5f). In addition, knockdown of HMGA1 reduced the expression of HMGA1P6 and HMGA2. Accordingly, knockdown of HMGA2 significantly suppressed HMGA1P6 and HMGA1 level (Fig. 5g). Furthermore, we performed RIP assay with anti-Ago2 antibody in HEY cells. As shown in Fig. 5h and Supplementary Fig. 2d, HMGA1P6 and miRNAs were enriched in Ago2-RIPs compared to control IgG-RIPs. Moreover, we conducted RNA pull down assay with HMGA1P6 bound magnetic beads. Western blot and mass spectrum (MS) analysis showed that Ago2 was pulled down by biotinylated HMGA1P6 (Fig. 5i and Supplementary Table 6). Functionally, the invasive capacity enhanced by HMGA1P6 overexpression was attenuated by HMGA1 silencing (Fig. 5j). Collectively, these results indicate that HMGA1P6 acts as a ceRNA to regulate HMGA1/2 expression in ovarian cancer.

### HMGA1P6 is transcriptionally activated by MYC in ovarian cancer

**Fig. 6:**
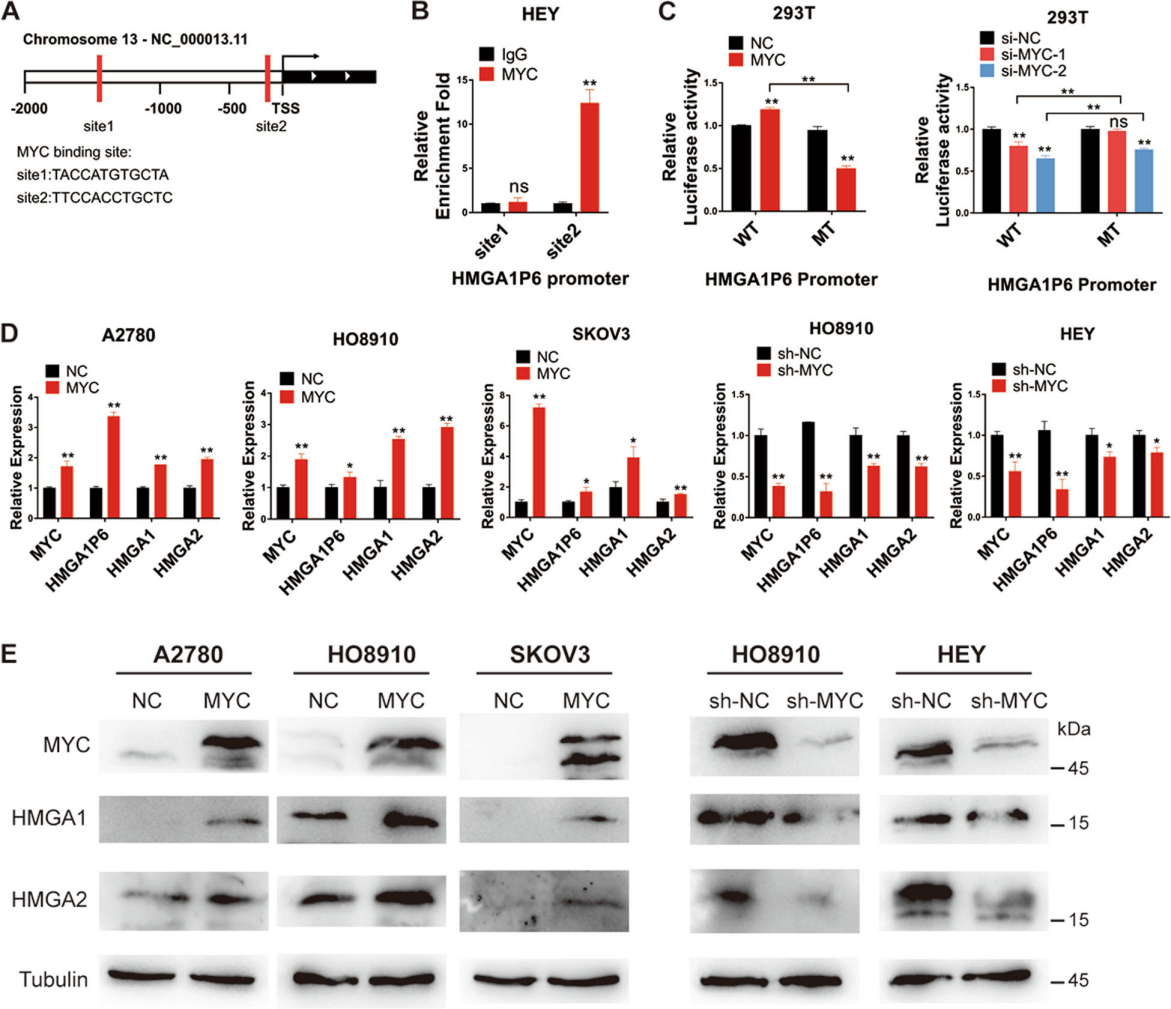
HMGA1P6 is transcriptionally activated by MYC in ovarian cancer. **a** Potential MYC binding sites were predicted on HMGA1P6 promoter region. **b** ChIP-qPCR was conducted to analyze the binding sites of MYC on promoter region of HMGA1P6 in HEY cells. **c** Luciferase activity was measured in HEK293T cells co-transfected with MYC overexpression plasmids, siRNAs and pGL4 plasmid containing HMGA1P6 promotor region (wild type and mutant). **d**, **e** MYC, HMGA1P6, and HMGA1/2 expression was measured in cells with MYC overexpression or knockdown. Data are presented as means ± S.D. *p < 0.05, **p < 0.01.

To explore the molecular mechanism involved in the upregulation of HMGA1P6 in ovarian cancer, HMGA1P6 promoter region was analyzed to search for potential transcriptional factors using online search website (http://jaspar.genereg.net/). MYC, which is commonly overexpressed in HGSOC and ovarian cancer cell lines (Supplementary Fig. 3a, b) was predicted with two binding sites on HMGA1P6 promoter region (Fig. 6a). Subsequently, we conducted ChIP assay and found MYC could directly bind HMGA1P6 promoter region with site2 but not site1 (Fig. 6b and Supplementary Fig. 3c). Luciferase assay was further performed and the result showed that forced expression of MYC increased while MYC knockdown reduced luciferase activity of HMGA1P6 promoter reporter carrying wild-type but not mutant MYC binding sites (Fig. 6c). We further measured HMGA1P6 and HMGA1/2 expression in ovarian cancer cell lines with MYC overexpression or knockdown. As illustrated in Fig. 6d, e, ectopic expression of MYC increased the expression of HMGA1P6 and HMGA1/2, while knockdown MYC declined the expression of HMGA1P6 and HMGA1/2. Moreover, we measured MYC, HMGA1P6, HMGA1, and HMGA2 expression by qPCR and found MYC expression level was correlated with HMGA1P6, HMGA1, and HMGA2 respectively (Supplementary Fig. 3d, e). Collectively, these data suggest that HMGA1P6 is a direct transcriptional target of MYC in addition to HMGA1 which has been previously reported^20^.

## Discussion

About 10% of human genes have their pseudogene counterparts^8^. Highly expressed housekeeping genes are more likely to produce pseudogenes^24^. RPs (Ribosomal proteins) have about 2000 pseudogenes in the human genome^25^. Pseudogenes have caught attention probably due to the finding that pseudogene PTENP1 can regulate its parent gene PTEN through ceRNA mechanism^10^. Pseudogenes are aberrantly expressed in a variety of cancer types and show cancer-specific expression^26^. It has been shown that pseudogenes are capable of regulating tumor suppressors and oncogenes by acting as microRNA decoys^7^. However, the expression pattern and the biological function of pseudogenes in ovarian cancer remains largely unknown. In this study, we analyzed global pseudogene expression pattern in HGSOC and found the majority of dysregulated pseudogenes were up-regulated (538 of 577). Hierarchical cluster and correlation matrix heat map analysis showed that HGSOC exhibited specific pseudogene expression pattern compared to normal tissues (Fig. 1a, b).

HMGA1P6 is a processed pseudogene of HMGA1 and produce a lncRNA (long noncoding RNA) because a mutation in termination codon^27^. HMGA1P6 is reported to be overexpressed in human pituitary and thyroid tumors, and regulates HMGA1 as a competing endogenous RNA^15,17^. Transgenic mice overexpressing HMGA1P6 develop lymphoma^28^. Our group systemically study the expression, function and upregulation mechanism of HMGA1P6 in HGSOC. We found HMGA1P6 is highly expressed in HGSOC and elevated level of HMGA1P6 is associated with poor prognosis. In addition, HMGA1P6 promotes ovarian cancer aggressiveness through modulating HMGA1 and HMGA2. Importantly, HMGA1P6 is a direct transcriptional target of MYC, which is frequently amplified in HGSOCs.

HMGA1 and HMGA2 are nonhistone chromatin remodeling proteins which contain AT-hook DNA binding domains^29^. Normally, HMGA1 and HMGA2 are expressed at high levels during embryogenesis, with low or absent levels in most adult tissues^29^. HMGA1/2 overexpression is a frequent event in human malignancies, and correlates with cancer progression, high aggressiveness, and poor prognosis^30^. The molecular mechanisms of HMGA1/2 reactivation in human malignancies have not been well characterized. HMGA1 is transcriptionally activated by MYC, MYCN, and AP-1^31–33^. Several miRNAs including miR-26a, let-7a modulate HMGA1 expression at post-transcriptional level^34,35^. HMGA2 is a notorious let-7 target associated with tumorigenesis^36^. We previously reported that HMGA2 overexpression is significantly associated with poor prognosis in patients with HGSOC^37^. A ceRNA mechanism is proposed for the increased HMGA1 expression. HMGA1P6 and HMGA1P7 regulate HMGA1 as competing endogenous RNAs in pituitary tumors^27^. Our study found that HMGA1P6 is overexpressed and acts as ceRNA of both HMGA1 and HMGA2 in HGSOC.

The MYC proto-oncogene is activated in more than half of all human cancers^38^. Over 30% of HGSOC exhibit MYC amplification (TCGA data). As a master transcriptional factor, MYC is able to modulate expression of a large number of target genes and regulate multiple cellular processes. MYC promotes tumorigenic immune evasion by inducing expression of CD47 and PD-L1^39^. LDH-A is a MYC-responsive target gene which is sufficient to induce the Warburg effect in human cancers^40^. Recent studies also suggest overexpression of MYC also induces widespread changes in non-coding transcription including lncRNA‐MIF and DANCR^38,41^. We here show that MYC transactivates a novel transcript, HMGA1P6 in ovarian cancer. To our knowledge, this is the first case in which MYC transcriptionally activated a pseudogene. Further investigation is needed to identify other pseudogene targets of MYC and determine their functions in cancer.

**Fig. 7:**
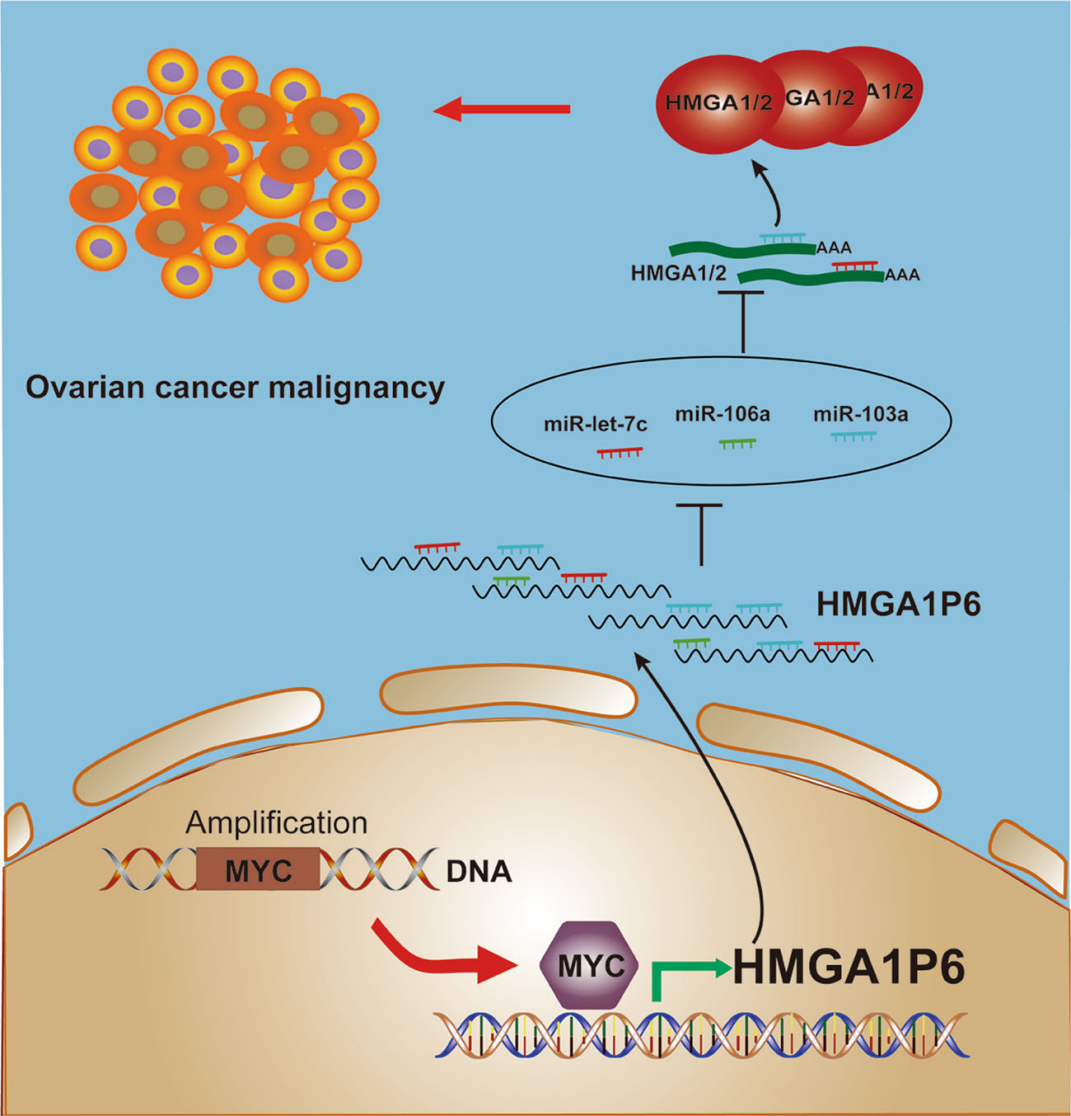
Proposed model of MYC activates HMGA1P6 in ovarian cancer. Oncogenic MYC transcriptionally activates pseudogene HMGA1P6 in HGSOCs. Ectopic expression of HMGA1P6 consequently augments the oncogenic HMGA1/2 acting as a competing endogenous RNA.

Taken together, our study characterized the pseudogene expression profile in HGSOC. We further demonstrated that pseudogene HMGA1P6 was overexpressed and its expression is correlated with poor prognosis in patients with HGSOC. Functional and mechanistic analysis revealed that HMGA1P6 increased malignancy by acting as a ceRNA that led to enhanced HMGA1/2 expression. Importantly, MYC transcriptionally upregulated HMGA1P6 as well as HMGA1^31^ in ovarian cancer. Although HMGA1P6, HMGA1, and HMGA2 were all overexpressed in HGSOC, only HMGA1P6 and HMGA2^37^ correlated with poor prognosis in cancer patients. Collectively, our findings suggest that pseudogene HMGA1P6 could be a valuable prognosis marker and promising therapy target for ovarian cancer. The proposed schematic model of MYC-HMGA1P6-HMGA1/2 in HGSOCs is summarized in Fig. 7.

## Supplementary information

Supplementary Figure 1

Supplementary Figure 2

Supplementary Figure 3

Supplementary Table 1

Supplementary Table 2

Supplementary Table 3

Supplementary Table 4

Supplementary Table 5

Supplementary Table 6

Supplementary Materials and Methods

Supplementary Figure and table legends

Supplementary western blot images
