# Supplementary figures and images for: MYC-regulated pseudogene HMGA1P6 promotes ovarian cancer malignancy via augmenting the oncogenic HMGA1/2
Source: Cell Death Dis. 2020 Mar 3;11(3):167. doi: 10.1038/s41419-020-2356-9 (PMC7054391; doi:10.1038/s41419-020-2356-9)

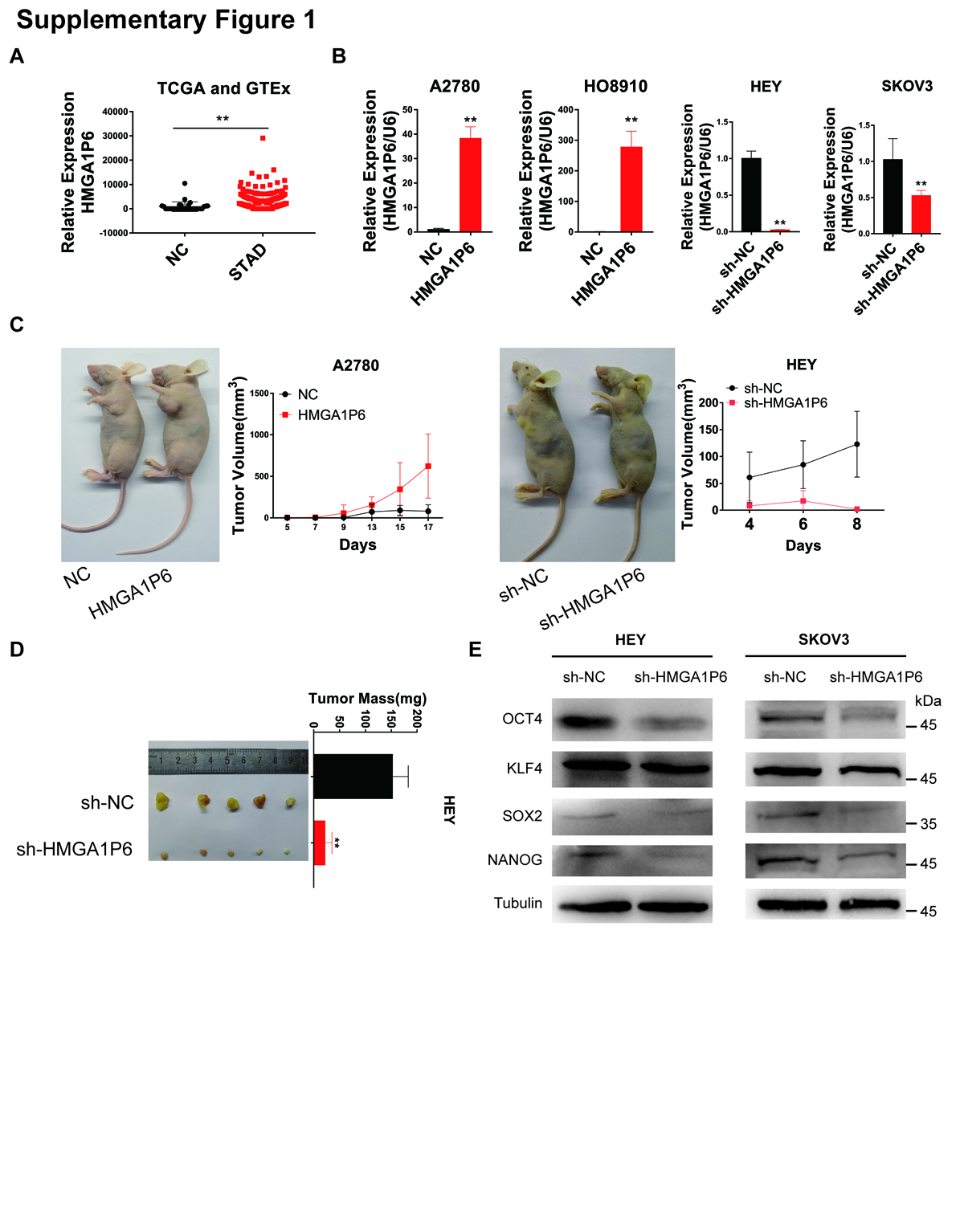

Supplement: Supplementary file 1 — Supplementary Figure 1 [file 41419_2020_2356_MOESM1_ESM.tif]

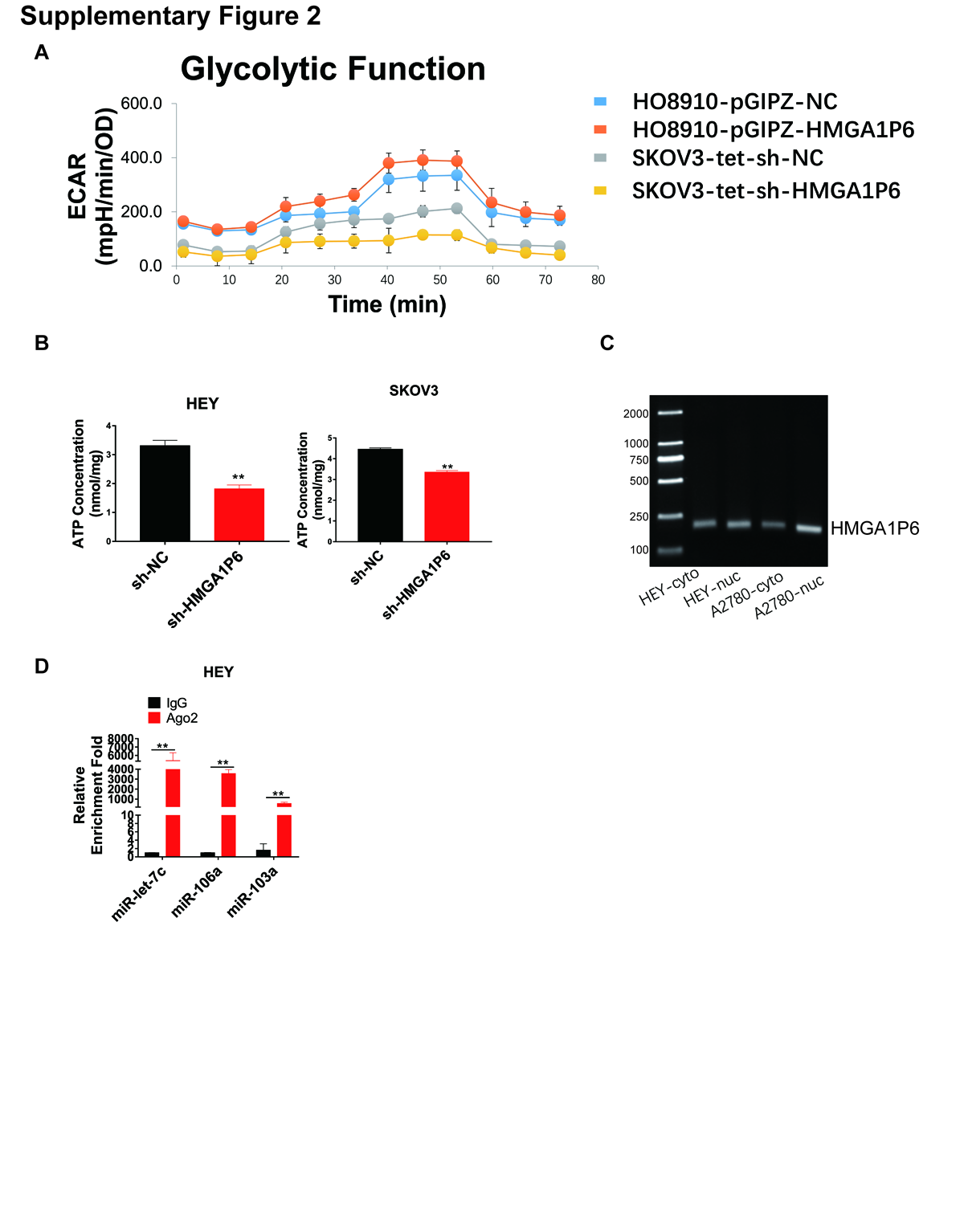

Supplement: Supplementary file 2 — Supplementary Figure 2 [file 41419_2020_2356_MOESM2_ESM.tif]

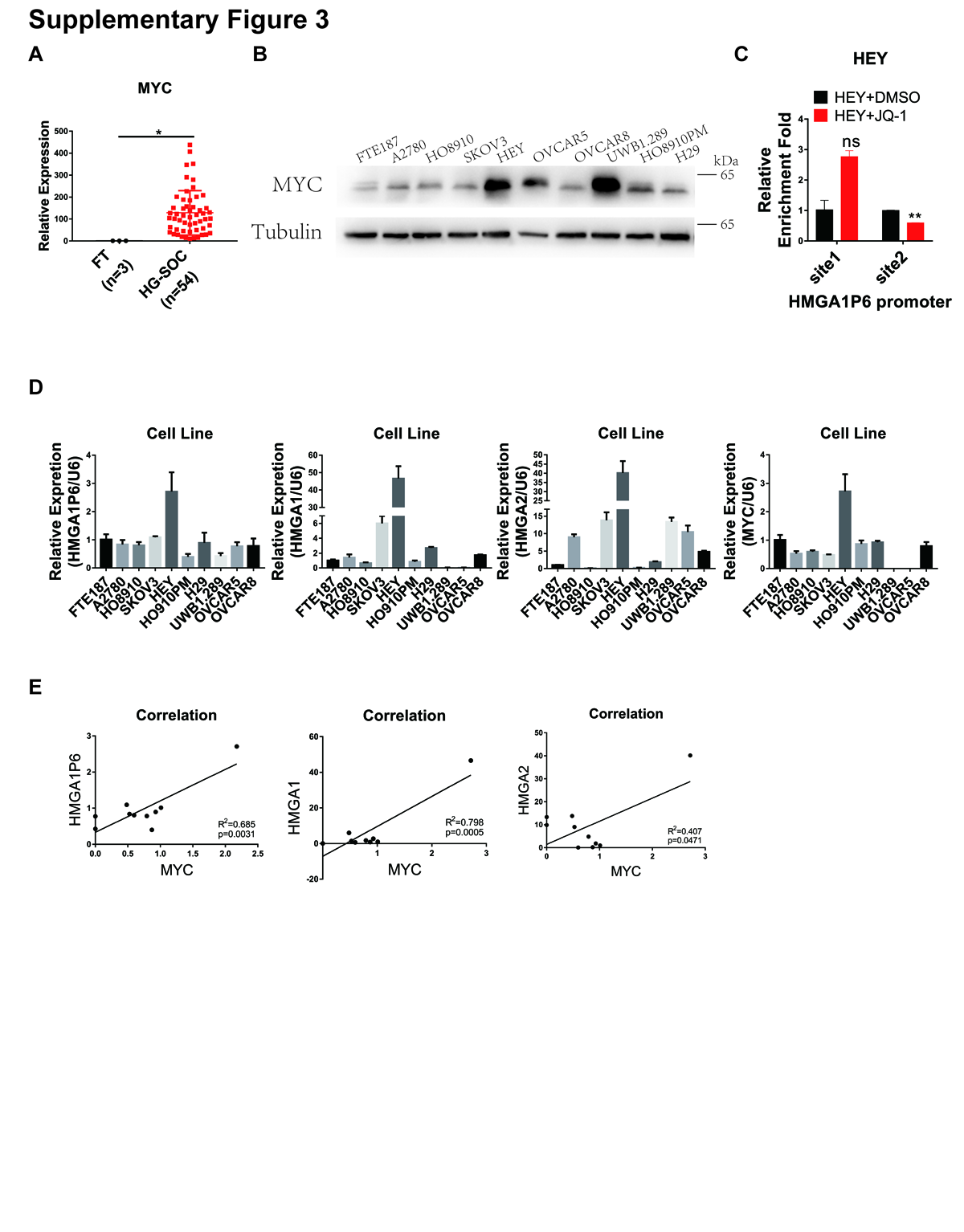

Supplement: Supplementary file 3 — Supplementary Figure 3 [file 41419_2020_2356_MOESM3_ESM.tif]
