# Supplementary material for: MYC-regulated pseudogene HMGA1P6 promotes ovarian cancer malignancy via augmenting the oncogenic HMGA1/2
Source: Cell Death Dis. 2020 Mar 3;11(3):167. doi: 10.1038/s41419-020-2356-9 (PMC7054391; doi:10.1038/s41419-020-2356-9)
Supplement: Supplementary file 4 — Supplementary Table 1 [file 41419_2020_2356_MOESM4_ESM.docx]

**Supplementary Table 1**

Primers information

| Method | Prime name | Primer sequence(5’-3’) |
| --- | --- | --- |
| qRT-PCR | HMGA1P6-F | \| GAGCGAGTTGAGCTTGAAGT \| \| --- \| |
| qRT-PCR | HMGA1P6-R | TTGTGATGGTTTTCTGGGACT |
| qRT-PCR | HMGB1L10-F | ATCCCTCCCAAAGGGGAGAC |
| qRT-PCR | HMGB1L10-R | GGACAGGCCAGGATGTTCTC |
| qRT-PCR | HMGN2L10-F | CCCTTTGGGTACCTTCTCTTCT |
| qRT-PCR | HMGN2L10-R | GCCAAGGTGAAGGACGAACC |
| qRT-PCR | HMGA1-F | CAACTCCAGGAAGGAAACCA |
| qRT-PCR | HMGA1-R | AGGACTCCTGCGAGATGC |
| qRT-PCR | HMGA2-F | GCAGCAGCAAGAACCAACC |
| qRT-PCR | HMGA2-R | TCTTGGCCGTTTTTCTCCAGT |
| qRT-PCR | miR-let-7c-5p-F | ACACTCCAGCTGGGTGAGGTAGTAGGTTGT |
| qRT-PCR | miR-106a-5p-F | ACACTCCAGCTGGGAAAAGTGCTTACAGTGC |
| qRT-PCR | miR-103a-3p-F | ACACTCCAGCTGGGAGCAGCATTGTACAGGG |
| qRT-PCR | HMGA1P6 promoter-site1-F | GAAGGTGACAGGGGCAAAAG |
| qRT-PCR | HMGA1P6 promoter-site1-R | AGGGAAGCCAGCTCTTTCAT |
| qRT-PCR | HMGA1P6 promoter-site2-F | TCCGAGCCAGGGCTACTTCTGGCGT |
| qRT-PCR | HMGA1P6 promoter-site2-R | GCCATCCTTTTCCCACTTGGAGGCC |
| qRT-PCR | U6-F | CTCGCTTCGGCAGCACA |
| qRT-PCR | U6-R | AACGCTTCACGAATTTGCGT |
| qRT-PCR | MYC-F | CTACCCTCTCAACGACAGCA |
| qRT-PCR | MYC-R | AGAGCAGAGAATCCGAGGAC |
