# Supplementary material for: MYC-regulated pseudogene HMGA1P6 promotes ovarian cancer malignancy via augmenting the oncogenic HMGA1/2
Source: Cell Death Dis. 2020 Mar 3;11(3):167. doi: 10.1038/s41419-020-2356-9 (PMC7054391; doi:10.1038/s41419-020-2356-9)
Supplement: Supplementary file 5 — Supplementary Table 2 [file 41419_2020_2356_MOESM5_ESM.docx]

**Supplementary Table 2**

Sequences of siRNAs, miRs-mimics and inhibitors

| si-MYC-1 | CGUCCAAGCAGAGGAGCAA |
| --- | --- |
| si-MYC-2 | CGAUGUUGUUUCUGUGGAA |
| si/sh-HMGA1P6 | GGCGUUGGCCCAGCUCCAA |
| sh-HMGA1 | ACAACTCCAGGAAGGAAACCAA |
| si-HMGA1 | CTCACCACCACACTACACA |
| si-HMGA2 | GCAGTGACCAGTTATTCTTTT |
| hsa-miR-let-7c-5p mimics | UGAGGUAGUAGGUUGUAUGGUU |
| hsa-miR-106a-5p mimics | AAAAGUGCUUACAGUGCAGGUAG |
| hsa-miR-103a-3p mimics | AGCAGCAUUGUACAGGGCUAUGA |
| hsa-miR-let-7c-5p inhibitor | AACCAUACAACCUACUACCUCA |
| hsa-miR-106a-5p inhibitor | CUACCUGCACUGUAAGCACUUUU |
| hsa-miR-103a-3p inhibitor | UCAUAGCCCUGUACAAUGCUGCU |
