# Supplementary material for: MYC-regulated pseudogene HMGA1P6 promotes ovarian cancer malignancy via augmenting the oncogenic HMGA1/2
Source: Cell Death Dis. 2020 Mar 3;11(3):167. doi: 10.1038/s41419-020-2356-9 (PMC7054391; doi:10.1038/s41419-020-2356-9)
Supplement: Supplementary file 6 — Supplementary Table 3 [file 41419_2020_2356_MOESM6_ESM.docx]

**Supplementary Table 3**

Primary antibody information

| Antibody | Company | Catalog number | Clone | Immune peptides |
| --- | --- | --- | --- | --- |
| HMGA1 | Abcam | ab4078 | polyclonal |  |
| HMGA2 | Sigma | SAB2701959 | polyclonal |  |
| ZO-1 | CST | 8193 | monoclonal | D7D12 |
| ZEB1 | CST | 3396 | monoclonal | D80D3 |
| E-Cadherin | ABclonal | A3044 | polyclonal |  |
| N-Cadherin | ABclonal | A3035 | polyclonal |  |
| β-Catenin | CST | 8480 | monoclonal | D10A8 |
| Vimentin | CST | 5741 | monoclonal | D21H3 |
| Snail | ABclonal | A5243 | polyclonal |  |
| Slug | ABclonal | A1057 | polyclonal |  |
| OCT4 | ABclonal | A7920 | polyclonal |  |
| MYC | CST | 9402 | polyclonal |  |
| KLF4 | ABclonal | A6640 | polyclonal |  |
| SOX2 | ABclonal | A0561 | polyclonal |  |
| NANOG | ABclonal | A3232 | polyclonal |  |
| Tubulin | Proteintech | 66031-1-Ig | monoclonal | 1E4C11 |
| Ago2 | Abcam | ab32381 | polyclonal |  |
| HuR | Abcam | ab136542 | monoclonal | 4C8 |
