# Supplementary material for: MYC-regulated pseudogene HMGA1P6 promotes ovarian cancer malignancy via augmenting the oncogenic HMGA1/2
Source: Cell Death Dis. 2020 Mar 3;11(3):167. doi: 10.1038/s41419-020-2356-9 (PMC7054391; doi:10.1038/s41419-020-2356-9)
Supplement: Supplementary file 8 — Supplementary Table 5 [file 41419_2020_2356_MOESM8_ESM.docx]

**Supplementary Table 5**

**Correlation between HMGA1P6 expression and clinicopathological features**

| **Clinicopathological features** | | **HMGA1P6 expression** | | **P-value** |
| --- | --- | --- | --- | --- |
|  |  | **Low expression** | **High expression** |  |
| Age | ＜50 | 8 | 2 | 0.0079 |
|  | ≥50 | 5 | 13 |  |
| FIGO staging | Ⅰ Ⅱ | 4 | 6 | 0.6188 |
|  | Ⅲ Ⅳ | 8 | 8 |  |
| CA125 | ＜500 | 4 | 7 | 0.3903 |
|  | ≥500 | 9 | 8 |  |
| Platinum status | Sensitive | 3 | 1 | 0.1709 |
|  | Resistance | 0 | 1 |  |
| Lymph node metastasis | Absent | 3 | 5 | 0.7373 |
|  | Present | 3 | 7 |  |
| Omentum metastasis | Absent | 6 | 6 | 0.5099 |
|  | Present | 4 | 7 |  |
