# Supplementary material for: MYC-regulated pseudogene HMGA1P6 promotes ovarian cancer malignancy via augmenting the oncogenic HMGA1/2
Source: Cell Death Dis. 2020 Mar 3;11(3):167. doi: 10.1038/s41419-020-2356-9 (PMC7054391; doi:10.1038/s41419-020-2356-9)
Supplement: Supplementary file 10 — Supplementary Materials and Methods [file 41419_2020_2356_MOESM10_ESM.docx]

**Supplementary Materials and Methods**

**Cellular energy metabolism analysis**

We used Seahorse metabolic analyzer and its matched Kit to complete this experiment. We used Seahorse XF Glycolysis Stress Test Kit to test the glycolytic function of cells. It measured glycolysis, glycolytic capacity and allow calculation of glycolytic reserve and non-glycolytic acidification utilizing glucose, oligomycin and 2-DG. All procedures were performed following manufacturer’s instructions. In general, we seeded cells in 96-well plate at about 1–1.5 × 10^4^ cells/well and hydrated a sensor cartridge at the day prior to assay. At the day of assay, we prepared assay medium according to kit instructions and washed cells with assay medium and prepared different compounds for assay. Then we used proper procedure to run assay and used MTT assay to normalize the data after the assay was done.

**ATP concentration analysis**

We used ATP Assay Kit (Beyotime Institute of Biotechnology, China) to complete this experiment and all procedures were performed following manufacturer’s instructions. Cells were cultured in low DMEM(1g/L) for 48h and then were lysed for ATP analysis. All samples were put on ice to avoid ATP degradation. ATP standard substance were used to make standard curve. All samples and standard substance were mixed with firefly luciferase substrate for luciferase activity detection witch could reflect ATP levels of cells.

**Nuclear and cytoplasmic RNA isolation assay**

We used PARIS Kit (Thermo Fisher Scientific, Waltham, MA, USA) to complete this experiment. In brief, first, we collected 10^2^–10^7^ HEY and A2780 cells and disrupted samples using Cell Fractionation Buffer. Then after centrifugation, the supernatant contained cytoplasmic RNA and the pellet contained nuclear RNA. At last, we collected nuclear and cytoplasmic RNA by loading corresponding lysate through filter cartridge.

**ChIP (Chromatin immunoprecipitation) assay**

ChIP assay was conducted using EZ-Magna ChIP A/G Chromatin Immunoprecipitation Kit (Merck KGaA, Darmstadt, Germany). HEY cells were preconditioned with DMSO/JQ-1 at 300nM over 72h and were cross-linked using 37% formaldehyde. Then the cells were lysed in lysis buffer and DNA was sheared to about 200-500bp fragments by sonication. The cell lysis was incubated with anti-MYC antibody and magnetic beads overnight at 4°C with rotation. DNA was purified and analyzed by qRT-PCR.

**miRNA First Strand cDNA Synthesis (Tailing Reaction) and qPCR**

miRNA RT-PCR was conducted using miRNA First Strand cDNA Synthesis (Tailing Reaction) (Sangon Biotech, Shanghai, China) following manufacturer’s instruction. Briefly, make a mixture like table below:

| 2×miRNA RT Solution mix | 10 µl |
| --- | --- |
| miRNA RT Enzyme mix | 2 µl |
| Total RNA | 2 µg |
| RNase-free water | up to 20 µl |

The mixture was incubated at 37℃ for 60min and then 85℃ for 5min. Then cDNA was diluted 10 times for next qPCR:

| 2×SYBR GREEN mix | 10 µl |
| --- | --- |
| miRNA-primer-F(10µM) | 0.4 µl |
| Universal- primer-R(10µM) | 0.4 µl |
| cDNA | 2 µl |
| RNase-free water | up to 20 µl |

Reaction condition was set according to SYBR GREEN mix instructions.
