# Supplementary material for: MYC-regulated pseudogene HMGA1P6 promotes ovarian cancer malignancy via augmenting the oncogenic HMGA1/2
Source: Cell Death Dis. 2020 Mar 3;11(3):167. doi: 10.1038/s41419-020-2356-9 (PMC7054391; doi:10.1038/s41419-020-2356-9)
Supplement: Supplementary file 11 — Supplementary Figure and table legends [file 41419_2020_2356_MOESM11_ESM.docx]

**Supplementary Figure legends**

**Supplementary Figure 1**

**a** TCGA data analysis of HMGA1P6 in stomach adenocarcinoma was conducted. **b** The overexpression and knockdown efficiency of HMGA1P6 were detected by qPCR. **c** Image of mice and the volumes of the tumors were recorded in xenograft model injected cells with HMGA1P6 overexpression or knockdown. **d** Xenografts of xenograft model injected cells with HMGA1P6 knockdown **e** Western blot analysis of stem cell markers in HMGA1P6 knockdown cells.

**Supplementary Figure 2**

**a** Glycolytic capacity was measured in cells with HMGA1P6 overexpression and knockdown using Seahorse metabolic analyzer and its matched kit **b** ATP production in HMGA1P6 knockdown cells were tested by ATP Assay Kit. **c** Nuclear and cytoplasmic RNA isolation assay was performed to analyze the subcellular localization of HMGA1P6 in HEY and A2780 cells. **d** Enrichment of miRNAs binding on Ago2 were detected by RIP assay with anti-Ago2 antibody in HEY cells.

**Supplementary Figure 3**

**a** Relative expression of MYC in HGSOC compared with FT was tested by qPCR. **b** expression of MYC in ovarian cancer cell lines in protein level was tested by western blot. **c** ChIP-qPCR of MYC binding sites at the promoter region of HMGA1P6 was conducted in HEY cells with or without JQ-1. **d** Relative expression of HMGA1P6, HMGA1, HMGA2 and MYC in ovarian cancer cell lines compared with FTE187 (normal fallopian tube cell line) was tested by qPCR. **e** Correlation analysis was conducted in cell lines between MYC and HMGA1P6, HMGA1, HMGA2.

**Supplementary Table legends**

**Supplementary Table 1**

Primers information used in this study.

**Supplementary Table 2**

Sequences of siRNAs, miRNA mimics and inhibitors used in this study.

**Supplementary Table 3**

Antibody information used in this study.

**Supplementary Table 4**

Transcriptome analysis of dysregulated pseudogenes in HGSOCs relative to FTs.

**Supplementary Table 5**

Association analysis between HMGA1P6 expression level and other clinicopathological factors.

**Supplementary Table 6**

Mass spectrum (MS) analysis of proteins pulled down by biotinylated HMGA1P6.
