# Supplementary material for: MYC-regulated pseudogene HMGA1P6 promotes ovarian cancer malignancy via augmenting the oncogenic HMGA1/2
Source: Cell Death Dis. 2020 Mar 3;11(3):167. doi: 10.1038/s41419-020-2356-9 (PMC7054391; doi:10.1038/s41419-020-2356-9)
Supplement: Supplementary file 12 — Supplementary western blot images [file 41419_2020_2356_MOESM12_ESM.docx]

**Western blot images related to Figure 3**

**HO8910**

**A2780**

**HMGA1P6**

**NC**

**HMGA1P6**

**NC**















**E-cadherin**

135

135

135

135

180

180

**ZEB1**

180

180







**N-cadherin**

65

65

100

100

**ZO-1**









**β-catenin**





**Vimentin**











**Slug**

**Snail**

25

25

25

45

25

45







**Tubulin**

**HEY**

**SKOV3**

**sh-NC**

**sh-HMGA1P6**

**sh-NC**

**sh-HMGA1P6**









65

65

100

100

135

135

135

135

**E-cadherin**

180

180

180

180





















**ZEB1**

**Vimentin**

**β-catenin**

25

25

**N-cadherin**

**ZO-1**









**Snail**

25

**Slug**

45









25

45

**Tubulin**

**Western blot images related to Figure 4**





**SKOV3**

**HEY**













45

15

15

15

45

**sh-HMGA1P6**

**sh-NC**

**sh-NC**

**sh-HMGA1P6**

15

**Tubulin**

**HMGA2**

**HMGA1**









**Tubulin**

**HMGA2**

**HMGA1**

45

15

15

HMGA1P6 μg 0 2 4

**Western blot images related to Figure 5**

**Positive Control**

**HMGA1P6**

**NC**

**Input**







35

**HUR**





100

**Ago2**

**Western blot images related to Figure 6**

**A2780**

**NC**

**MYC**

**NC**

**MYC**

**SKOV3**

**HO8910**

**NC**

**MYC**



























45

45

45

15

15

15

15

15

15

**Tubulin**

**HMGA2**

**HMGA1**

65

65

65

**MYC**

**HEY**

**sh-MYC**

**MYC**

**HMGA1**

**Tubulin**

**HO8910**

**HMGA2**

**sh-NC**











15

65

65

15

**sh-MYC**

**sh-NC**







15

15







45

45

**Western blot images related to Supplementary Figure 1**























**sh-NC**

**sh-HMGA1P6**

35

65

45

45

65

45

45

**SKOV3**

**sh-HMGA1P6**

**KLF4**

**SOX2**

**NANOG**

**OCT4**

**HEY**

**Tubulin**

**sh-NC**

**sh-HMGA1**

**Western blot images related to Supplementary Figure 3**





HO8910PM

OV90

OVCAR3

OVCAR8

OVCAR5

HEY

HO8910

SKOV3

A2780

FTE187

H29

UWB1.289

**MYC**

**Tubulin**
